# The Childbirth Experience Questionnaire (CEQ)—Validation of its use in a Danish-speaking population of new mothers stimulated with oxytocin during labour

**DOI:** 10.1371/journal.pone.0233122

**Published:** 2020-05-14

**Authors:** Sidsel Boie, Henrik Hein Lauridsen, Julie Glavind, Mette Kiel Smed, Niels Uldbjerg, Pinar Bor

**Affiliations:** 1 Department of Obstetrics and Gynaecology, Regional Hospital of Randers, Randers NØ, Denmark; 2 Department of Sports Science and Clinical Biomechanics, University of Southern Denmark, Odense, Denmark; 3 Department of Obstetrics and Gynaecology, Aarhus University Hospital, Aarhus, Denmark; 4 Department of Obstetrics and Gynaecology, Rigshospitalet, Copenhagen, Denmark; Chinese Academy of Medical Sciences and Peking Union Medical College, CHINA

## Abstract

**Background:**

When determining optimal treatment regimens, patient reported outcomes including satisfaction are increasingly appreciated. It is well established that the birth experience may affect the postnatal attachment to the newborn and the management of subsequent pregnancies and deliveries. As we have no robust validated Danish tool to evaluate the childbirth experience exists, we aimed to perform a transcultural adaptation of the Childbirth Experience Questionnaire (CEQ) to a Danish context.

**Methods:**

In accordance with the COnsensus-based Standards for the selection of health Measurement INstruments (COSMIN), we translated the Swedish-CEQ to Danish. The Danish-CEQ was tested for content validity among 10 new mothers. In a population of women who have had their labour induced, we then assessed the electronic questionnaire for validity and reliability using factor analytical design, hypothesis testing, and internal consistency. Based on these data, we determined criterion and construct responsiveness in addition to floor and ceiling effects.

**Results:**

The content validation resulted in minor adjustments in two items. This improved the comprehensibility. The electronic questionnaire was completed by 377 of 495 women (76.2%). The original Swedish-CEQ was four-dimensional, however an exploratory factor analysis revealed a three-dimensional structure in our Danish population (Own capacity, Participation, and Professional support). Parous women, women who delivered vaginally, and women with a labour duration <12 hours had a higher score in each domain. The internal consistency (Cronbach’s alpha) ranged between 0.75 and 0.89 and the ICC between 0.68–0.93. We found ceiling effects of 57.6% in the domain Professional support and of 25.5% in the domain Participation.

**Conclusion:**

This study offers transcultural adaptation of the Swedish-CEQ to a Danish context. The 3-dimensional Danish-CEQ demonstrates construct validity and reliability. Our results revealed significant ceiling effect especially in the domain Professional support, which needs to be acknowledged when considering implementing the Danish-CEQ into trials and clinical practice.

## Background

Patient reported outcomes such as satisfaction are increasingly appreciated to be an important secondary outcome when determining optimal treatment regimens [1, 2, 3]. It is well established that the birth experience has an impact on subsequent pregnancy and birth in regard to the interval between pregnancies [4] and/or a demand for elective caesarean section [5, 6]. Moreover, the birth experience is also correlated to the risk of postpartum depression [7]. It is therefore important to be able to identify women reporting a negative birth experience to enable further and more intensive postnatal care and/or subsequent prenatal care.

Several tools for evaluating childbirth experience have been developed[8]. To our knowledge the Childbirth Experience Questionnaire (CEQ) is the only instrument that provides valid scores and comprehensively evaluates women’s perceptions and feelings [8]. It was developed to study women’s perceptions of their first labour and reported by Dencker et al in 2010 [9]. The Swedish version of the CEQ has been demonstrated to be a sufficiently reliable and valid tool to evaluate multidimensional aspects of nulliparous women’s experience of childbirth, including their perceptions and feelings [8]. The CEQ has been translated to several languages; e.g. English [10], and Spanish [11]. The Spanish version of the CEQ was assessed and validated in a population of both nulliparous and parous women.

In the absence of a robust, validated Danish tool for measuring birth experience we aimed to translate and test the Childbirth Experience Questionnaire for item characteristics, validity, reliability and floor/ceiling effects in a Danish speaking population and thereby perform a transcultural adaptation of the CEQ.

## Methods

### Translation procedure

Since Swedish and Danish are quite similar, the original CEQ was translated in 2014 using the simplified method of Beaton et al [12]. Two bilingual (Swedish-Danish) translators separately carried out the translation from Swedish to Danish. A group consisting of two research midwives synthesized the translations into one version. Another bilingual translator, blinded to the original Swedish version, translated the Danish version back into Swedish. Each item from the forward-backward translation was contrasted with the original, consensus was made on all differences, and the final result was documented. In cases where the terminology required clarification (e.g. differences between ‘delivery’ and ‘birth’), the first author of the original Swedish questionnaire was consulted. Translators and researchers finally agreed on a preliminary version of the Danish CEQ. In 2015 we conducted interviews with ten postpartum women to test feasibility, relevance, comprehensiveness and comprehensibility of the preliminary Danish CEQ [13].

### The validation study

#### Setting and participants

The sample included women participating in a study on induced labour and the use of oxytocin in Randers Regional Hospital, Aarhus University Hospital, Aalborg University Hospital, Sygehus Lillebaelt—Kolding, Herning Regional Hospital, Rigshospitalet, Nordsjaellands Hospital and Odense University Hospital in the period April 2016 through December 2018.

The included women were 18 or more years old, with a singleton pregnancy at term with a cephalic presentation, intended vaginal delivery, and stimulated with oxytocin in the latent phase of labour (cervical orifice ≤ 4 cm) in order to induce labour. Exclusion criteria was an estimated fetal weight >4500 g, an abnormal CTG prior to stimulation with oxytocin, or an inability to read and understand Danish.

All the eligible women received written and oral information about the study methods, the aims of the research and the possible adverse events related to the interventions, and gave written informed consent. In accordance with Danish legislation, the consent forms will be kept safe until 5 years after completion of the study.

#### Procedure

Demographic baseline data on pregnancy and delivery outcome were collected on all participants. The Danish version of the CEQ was sent to all participants by e-mail four weeks postnatally using REDcap (Research Electronic Data Capture) electronic data capture tools hosted at Aarhus University [14]. REDCap is a secure, web-based application designed to support data capture for research studies, providing 1) an intuitive interface for validated data entry; 2) audit trails for tracking data manipulation and export procedures; 3) automated export procedures for seamless data downloads to common statistical packages; and 4) procedures for importing data from external sources.

Reminders were sent twice, after one and two days, to those who had not replied to the primary request. Women enrolled during the period June 2018 to November 2018 were requested to complete the questionnaire twice, at four and six weeks post partum.

### The Childbirth Experience Questionnaire (CEQ)

The original CEQ was developed using a Swedish population of nulliparous pregnant women participating in a trial on labour and oxytocin augmentation [9]. The first author of this publication gave us permission to reproduce the CEQ which covers multidimensional aspects comprehensively exploring women’s perceptions of and feelings about their first labour and birth. The CEQ contains 22 statements assessing four domains of the childbirth experience; Own capacity, Professional support, Participation, and Perceived safety. For 19 of the items the response format is a 4-point Likert Scale whereas the last three items use a visual analogue scale (VAS).

The scoring range is 1 to 4 where higher ratings reflect more positive experiences. Item ratings are aggregated to domain scores by summing the coded response values of the items in each domain and dividing by the number of items answered in that domain to derive a mean value. The values are only considered valid if at least half the responses have been complete. The authors of the original Swedish CEQ developed instructions on item scoring and these instructions were transferred to the Danish version of the CEQ (S1 Table). It should take the woman only five to ten minutes to complete the questionnaire.

### Statistics and analyses

Measurement properties of the Danish version of the CEQ were evaluated using the COSMIN taxonomy and methodology [15].

Baseline characteristics with a normal distribution are presented with a mean and SD whereas non-normally distributed characteristics are presented using median and interquartile range (IQR). All the analyses were performed using Stata Version 14.

#### Item characteristics

Item characteristics were calculated including the percentage of missing items, skewness, and kurtosis. Items were considered valid if the response rate was at least 97%. A kurtosis of three demonstrates normal distribution [16]. A kurtosis below three is defined as platykurtosis where the distribution produces fewer and less extreme outliers than does the normal distribution. A kurtosis above three is defined as leptokurtosis which has fatter tails and produces more extreme values compared to normal distribution [16].

A skewness of zero corresponds to normal distribution; if the skewness is below zero will the distribution be skewed to the left, indicating that the mean is lower than the median.

#### Structural validity

Exploratory Factor Analysis (EFA) was carried out on a sample of 348 responders with a responder:item ratio of 16:1 using a stepwise approach [17]. Sampling adequacy was tested using Bartlett’s Test of Sphericity and the Kaiser–Meyer–Olkin (KMO) test, and by scrutinizing the correlation matrix[18]. We chose principal axis factor analysis using a polychoric correlation matrix because the primary goal was to identify latent dimensions and since the items of the CEQ are categorical [17]. This was combined with oblique oblimin rotation with Kaiser normalization to obtain more meaningful and correlated factors. The number of factors to extract was examined using eigenvalues > 1, examination of screeplot, and parallel analysis [19]. Finally, we used a stepwise approach to improve the factor loadings and communalities. Factor loadings of > 0.7 and communality of > 0.5 were considered satisfactory [19].

#### Internal consistency

Internal Consistency is a measure of the interrelatedness among the items of a one-dimensional scale [15]. Internal consistency was measured by calculating Cronbach’s alpha for each of the domains. A Cronbach’s alpha of 0.7–0.9 was regarded as satisfactory.

#### Reliability

To assess reliability, we adopted a test-retest design with a two-week interval. We assumed that the experience of responders during that period was constant. Both scores were used to calculate the absolute score difference for each of the 22 items of the questionnaire. As the score of the items are aggregated score of each domain, they can be accepted as continuous. We therefore present an ICC (2,1) (Intraclass Correlation Coefficient) for each domain and for the total score. ICC > 0.7 are considered acceptable [20].

#### Construct validity–known group validation

The construct validity of the Danish CEQ was measured with the method of known-groups validation. Known-groups validation assessed the ability of the CEQ to distinguish between subgroups expected to differ on key sociodemographic or clinical variables. A comparison was made of CEQ domain scores (average of the individual domain scores) for nulliparous women aged < 35 years versus nulliparous women aged > 35 years [21], women who delivered vaginally versus those who had an instrumental delivery (caesarean or forceps) [22], and nulliparous versus parous [23]. Some of the comparison groups were identical with those used in the validation study of Swedish women [9] and the study on women in the United Kingdom (UK). Data were tested for normality of distribution by the Mann Whitney *U* test to compare domain scores between the groups.

#### Floor and ceiling effect

Floor and ceiling effects can often occur when an existing instrument is applied in a new target population [15]. Ceiling effect is when the responders with highest score in a domain are unable score any higher. This is especially a problem in follow up studies where responders then cannot score higher in the next survey. No more than 15% of the scores should be at the low or upper end of the 4-point Likert scale for each domain and for the overall score. If more than 15% of the scores are in the upper or lower end of a domain or the total score (ceiling or floor effect), the questionnaire is not able to differentiate between the scores, indicating that the score scale is not suitable.

The study was approved by The Danish Data Protection Agency (1-16-02-398-15). Questionnaire studies do not need approval by The Central Denmark Region Committee on Biomedical Research Ethics. However, The Central Denmark Region Committee on Biomedical Research Ethics and The Danish Medicine Agency approved the trial on labour induction and oxytocin usage, Eudra CT 2015-002942-30.

## Results

### Translation

The interviews resulted in minor adjustments in two items (item 4 and item 18) to improve comprehensibility. In item 4 “I felt skilled…” (“Jeg var dygtig…”) was changed to “I felt able to give birth” (“Jeg følte mig i stand til at gennemføre fødslen”) and in item 18 “medical competences” (“medicinske kompetencer”) was changed to “the competences of the staff” (“personalets faglige kompetencer”). The researchers and first author of the original Swedish questionnaire agreed on the final Danish version, Table 1.

**Table 1:** The final version of the Danish Childbirth Experience Questionnaire, CEQ.

Item	Domain	Statement
1	Own capacity	Fødslen forløb som jeg havde forestillet mig
2	Own capacity	Jeg følte mig stærk under fødslen.
3	Own capacity	Jeg følte mig bange under fødslen.
4	Own capacity	Jeg følte mig i stand til at gennemføre fødslen.
5	Own capacity	Jeg var træt under fødslen.
6	Own capacity	Jeg var glad under fødslen
7	Own capacity	Jeg har mange positive minder fra fødslen
8	Own capacity	Jeg har mange negative minder fra fødslen
9	Own capacity	En del af minderne fra fødslen kan få mig til at føle mig nedtrykt
10	Participation	Jeg følte, at jeg havde mulighed for at påvirke, om jeg skulle være oppe og røre mig eller ligge ned
11	Participation	Jeg følte, at jeg havde mulighed for at påvirke fødselsstillingen
12	Participation	Jeg følte, at jeg havde mulighed for at påvirke valg af smertelindring
13	Professional support	Jordemoderen brugte tilstrækkelig tid på mig
14	Professional support	Jordemoderen brugte tilstrækkelig tid på min partner
15	Professional support	Jordemoderen informerede om, hvad der skete under fødslen
16	Professional support	Jordemoderen forstod mine behov
17	Professional support	Jeg følte, at jordemoderen behandlede mig godt
18	Professional support	Mit indtryk af sundhedspersonalets faglige kompetence gjorde mig tryg
19	Own capacity	Jeg følte, at jeg håndterede situationen godt
20	Own capacity	Hvor smertefuldt oplevede du generelt fødslen^1^
21	Own capacity	Hvor meget kontrol følte du, at du generelt havde under fødslen?^1^
22	Own capacity	Når du tænker tilbage på fødslen, hvor tryg følte du dig generelt^1^

1. Reported on a VAS scale, changed for categorical values:0–40 = 1, 41–60 = 2, 61–80 = 3 and 81–100 = 4

During the interviews and the validation process our attention was drawn to the first item of the CEQ, “The labour progress went as I had expected” (“Fødslen forløb som jeg havde forestillet mig”). Expectations can be either negative or positive, which the response does not reflect. Despite the potential issues, we decided to retain the item since it is included in all other translated versions of the CEQ.

### Validation

We identified 505 women who all agreed to join the study between April 2016 and December 2018. We couldn’t read the handwritten e-mail address of 10 of the women. Four-hundred-and-ninety-five women received an electronic questionnaire. Of these, 377 women completed the first questionnaire (76.2% response rate). Of the 79 women requested to answer the CEQ twice, 57 women completed both (72.2% response rate). The characteristics of the study population are shown in Table 2.

**Table 2:** Baseline characteristics of the study population.

	Study population		
Demographic characteristics	Responders (n = 377)	Non-responders (n = 128)	*P-value*
Maternal age, mean (SD)	31.2 years (4.55)	30.7 (5.35)	0.36
Gestational age, median (IQR)	284 (275;291)	284 (276;291)	0.89
Nulliparous, n(%)	258 (68%)	84 (66%)	0.56
Vaginal delivery, n(%)	280 (75%)*	89 (72%)**	0.44
Instrumental delivery, n(%)	38(10%)	12 (10%)	0.86
Caesarean section, n(%)	54 (15%)	23 (18%)	0.28
BMI, median (IQR)	25.2 (22.3;29.7)**	24.7 (22.6;29.0)**	0.93
Epidural use	218 (57.8%)***	85 (66.4%)***	0.04
PPH > 500 ml	165 (43.8%)	61 (47.6%)	0.09
Labour duration > 12 hours	102 (36%)	46 (35.9%)	0.06
Umbilical pH<7.1	19 (5.0%)	3 (2.3%)	0.19
Apgar score <7 at 5 minutes	1 (0.27%)	2 (1.1%)	0.10
Perineal tear III or IV	15 (4.0%)	6 (4.7%)	0.19

* 5 missing values

** 4 missing values

*** 2 missing values

#### Item characteristics

Table 3 shows the characteristics for each item.

Overall there was very little missing data. The responses from two participants were excluded from the analysis of one domain each (one in Professional Support and one in Participation) due to a high number of missing responses (in five of six items and in two of three items, respectively). For ten (9, 12,13,14,15,16,17,18,19, and 22) of the 22 items of the CEQ the median score was 4, the highest possible score on the Likert scale. In three items (13, 17, and 18) we found a large kurtosis and a skewness indicating that the scores for these items are not normal distributed.

**Table 3:** Item characteristics.

Item	Mean (SD)	Median	Missing (%)	Skewness**	Kurtosis*
1	2.34 (1.0)	3	2 (0,54%)	-0,98	1.80
2	2.92 (0.84)	3	0	-0.58	2.90
3	3.18 (0.93)	3	1 (0.27%)	-0.70	2.28
4	3.19 (0.86)	3	1 (0.27%)	-0.96	3.35
5	1.98 (1.02)	2	0	0.78	2.49
6	2.84 (0.84)	3	1 (0.27%)	-0.56	2.91
7	3.08 (0.91)	3	1 (0.27%)	-0.63	2.41
8	2.95 (0.94)	3	1 (0.27%)	-0.32	1.96
9	3.39 (0.93)	4	1 (0.27%)	-1.30	3.46
10	3.06 (1.07)	3	3 (0.81%)	-0.78	2.26
11	2.84 (1.12)	3	7 (1.89%)	-0.46	1.83
12	3.47 (0.83)	4	4 (1.08%)	-1.52	4.46
13	3.87 (0.43)	4	1 (0.27%)	-3.74	18.55
14	3.78 (0.50)	4	5 (1.32%)	-2.35	8.44
15	3.74 (0.56)	4	1 (0.26%)	-2.48	9.96
16	3.71 (0.57)	4	1 (0.26%)	-2.17	8.08
17	3.90 (0.38)	4	1 (0.26%)	-4.32	23.20
18	3.82 (0.45)	4	1 (0.26%)	-2.67	10.67
19	3.47 (0.66)	4	1 (0.26%)	-1.21	4.55
20	1.59 (0.82)	1	3 (0.81%)	1.36	4.20
21	2.56 (1.12)	3	3 (0.81%)	-0.11	1.64
22	3.56 (0.84)	4	0	-1.83	5.27
Total	3.14	3.23			

* Kurtosis: 3 = normal distribution, >3 = leptokurtic, produces more outliers than normal distribution, and <3 = platykurtic, produces fewer and less extreme outliers than normal distribution[16]

**Skewness 0 = normal distribution <0 = the distribution is skewed to the left, the mean is lower than the median

#### Construct validity—exploratory factor analysis

The data from 348 participants who responded to all items in the CEQ were included in this analysis. Tests for sampling adequacy showed that the sample was factorable (Bartlett’s test of sphericity had a p<0.001, the KMO was 0.907, and no item correlations >0.8 were identified) where the CEQ either had a 3-factor (eigenvalue > 1; parallel analysis; Velicer’s minimum average partial) or a 4-factor structure (screeplot)

We therefore analysed both three and a four factor models. We decided to perform oblique rotation with Horst normalization, as we expected the factors to be correlated.

The four-factor model showed several cross loadings, item 5 and 12 had communalities <0.4, and only nine items had a factor loading >0.7, Table 4.

**Table 4:** Factor loadings, communality, eigenvalues, explained variance, and Cronbach’s a of 3 and 4-solutions after exploratory factor analysis with rotation.

	4 factor model					3 factor models											
	All items					All items				21 items (item 5 deleted)				20 items (item 5 and 12 deleted)			
Item number and content	1	2	3	4	Com*	1	2	3	Com*	1	2	3	Com*	1	2	3	Com*
1 The labour progress went as I had expected	0.45				0.46	0.52			0.46	0.51			0.46	0.67			0.48
2 I felt strong	0.64				0.63	0.77			0.62	0.76			0.62	0.70	0.35		0.61
3 I felt scared	0.73			0.33	0.50	0.62			0.44	0.59			0.42	0.59			0.42
4 I felt capable	0.58				0.65	0.80			0.65	0.77			0.62	0.68	0.38		0.61
5 I felt tired	0.37				0.22	0.39			0.22	-	-	-	-	-	-	-	-
6 I felt happy	0.45			0.43	0.59	0.75			0.56	0.75			0.57	0.67	0.36		0.58
7 I have many positive memories from the labour process	0.70				0.77	0.79			0.77	0.78			0.78	0.84			0.77
8 I have many negative memories from the labour process	0.66				0.73	0.71			0.72	0.70			0.71	0.82			0.71
9 Some of my memories from the labour process make me feel depressed	0.60				0.61	0.62			0.60	0.60			0.60	0.77			0.60
10 I felt I could choose whether I should be up and moving or lie down			0.93		0.87			0.92	0.86			0.90	0.83	0.54	0.31	0.66	0.82
11 I felt I could choose the delivery position			0.88		0.80			0.86	0.79			0.86	0.78	0.55		0.65	0.81
12 I felt I could choose which pain relief method to use			0.44		0.37			0.44	0.36			0.43	0.36	-	-	-	-
13 My midwife devoted enough time to me		0.82			0.88		0.78		0.83		0.79		0.84	0.75	0.50		0.84
14 My midwife also devoted enough time to my partner		0.79			0.66		0.75		0.63		0.74		0.61	0.60	0.47		0.61
15 My midwife kept me informed about what was happening during labour and birth		0.83			0.80		0.79		0.76		0.80		0.76	0.71	0.46		0.76
16 My midwife understood my needs		0.72			0.83		0.71		0.81		0.72		0.82	0.78	0.43		0.81
17 I felt very well taken care of by the midwife	0.61	0.55		0.35	0.92		0.73		0.82		0.75		0.83	0.81	0.36		0.83
18 My impression of the medical competence made me feel secure		0.53			0.50		0.56		0.50		0.54		0.49	0.65			0.49
19 I felt that I handled the situation well	0.49				0.49	0.68			0.49	0.68			0.50	0.62	0.31		0.48
20 Experienced level of labour pain, VAS**				0.96	0.89	0.73			0.45	0.72			0.44	0.43	0.52		0.46
21 Experienced level of control, VAS**	0.55		0.30		0.59	0.68			0.59	0.67			0.58	0.69			0.59
22 Experienced level of sense of security; VAS^1^	0.86				0.86	0.66	0.31		0.75	0.65	0.33		0.75	0.86			0.75
Eigenvalue	10.11	2.34	1.24	0.92		10.11	2.34	1.24		9.89	2.29	1.19		9.64	2.25	1.14	
Variance %	43.1%	31.5%	24.7%	17.9%		43.9%	32.5%	22.6%		45.7%	36.3%	24.4%		46.8%	36%	22.7%	
Crohnbach’s Alpha	0.87	0.85	0.75	0.67		0.89	0.85	0.75		0.90	0.85	0.75		0.90	0.85	0.85	

*Communality

**VAS-scales scores adapted to categorical values: 0–40 = 1, 41–60 = 2, 61–80 = 3, and 81–100 = 4

We therefore discarded the 4-factor solution.

Three different models for a 3-factor solution were tested. The full version, 22-item 3-factor solution, showed poor factor loadings and communalities of items 5 and 12. The 21-item 3 factor solution deleted the poorest fitted item, resulting in a further reduction of factor loading for item 12. The last model deleted both 5 and 12, resulting in too few items to form a third factor [24]. However, both item 5 and 12 were regarded as clinical important items.

We therefore accepted the 22-item 3-factor solution as the best option despite the poor loading on item 5 and 12. The factors were labelled “Own capacity“(factor 1), “Professional support” (factor 2), and “Participation” (factor 3) in an attempt to use the original Swedish terminology.

Due to the earlier mentioned issues concerning item 1 we performed and additional analysis excluding this item. However, no substantial differences were observed; hence we accepted inclusion of item 1.

#### Internal consistency

Cronbach’s alpha was between 0.75 and 0.89 for the three domains (Own capacity 0.89, Professional support 0.85, and Participation 0.75), Table 4 reflecting a high internal consistency.

#### Reliability

Fifty-seven of 79 invited women completed both the first and second CEQ. For these participants the Interclass correlation coefficient (ICC 2,1) for each domain of the CEQ and for the overall CEQ score were calculated and presented in Table 5.

**Table 5:** Test-retest reliability for the domains of the CEQ, the quadratic weighted index of agreement (weighted kappa).

Domain	ICC (2,1)
Own capacity	0.93
Professional support	0.86
Participation	0.68
Total score	0.92

The ICC was ≥ 0.7 for Own capacity (0.93), Professional Support (0.86) and the total score (0.92), but for Participation we only found an ICC of 0.68.

#### Construct validity–known-groups validation

Construct validity of the CEQ was measured using the methods of known-groups validation as shown in Table 6.

**Table 6:** Differences in subscale scores and overall score of the CEQ by different groups.

Subgroup	Own capacity	Professional Support	Participation	Overall score
Nulliparous n = 258	2.79	3.78	3.03	3.09
Parous n = 119	3.00	3.85	3.31	3.27
Unadjusted p value	0.002	0.11	0.002	<0.001
Adjusted p value (bonferroni)	0.001	0.18	0.003	0.001
Vaginal delivery, n = 280	2.98	3.84	3.19	3.25
Operative delivery*, n = 92	2.47	3.67	2.89	2.85
Unadjusted p value	<0.001	<0.001	0.002	<0.001
Adjusted p value (bonferroni)	<0.001	<0.001	0.003	<0.001
Labour duration <12 hours, n = 275	2.94	3.83	3.18	3.22
Labour duration >12 hours, n = 102	2.61	3.72	2.97	2.95
Unadjusted p value	<0.001	0.015	0.03	<0.001
Adjusted p value (bonferroni)	<0.001	0.021	0.03	<0.001
Nulliparous >35 years old, n = 48	2.73	3.75	3.00	3.05
Nulliparous <35 years old, n = 210	2.80	3.79	3.04	3.10
Unadjusted p value	0.49	0.55	0.82	0.52
Adjusted p value (bonferroni)	0.53	0.55	0.81	0.58

*Operative delivery includes vacuum extraction and caesarean section

Eleven out of 16 pre-specified hypotheses were accepted.

Parous women were significantly more likely to have higher scores for the following domains: Own capacity p<0.001) and Participation (p = 0.002), compared to nulliparous. There was no statistically significant difference between scores for the domain Professional support (p = 0.11).

Women who had a vaginal delivery were significantly more likely to have higher scores for all domains of the CEQ (Own capacity (p<0.001), Professional support (p<0.001), and Participation (p = 0.002)) than women who were delivered by caesarean or instrumentally. There was no statistically significant difference between scores for any domains, or the total score, when comparing nulliparous women aged <35 year old to nulliparous >35 years old, but in all domains the score tended to be lower in women above 35 years of age.

#### Floor and ceiling effect

Floor and ceiling effects are demonstrated in Table 7.

**Table 7:** Floor and Ceiling effects for each domain of the CEQ and for the total score.

Domain	Floor effect n(%)	Ceiling effectn(%)
Own capacity	1 (0.27%)	1 (0.27%)
Professional support	-	217 (57.6%)
Participation	8 (2.2%)	100 (26.5%)
Overall score	-	-

We found a high ceiling effect in two domains; Professional support (57.6%) and Participation (26.5%) suggesting that scores in these domains, especially professional support, have difficulties in discriminating the experience between responders.

## Discussion

To our knowledge this is the first validated birth experience tool in Danish. Our methods are sound and thorough and in line with how to perform validation studies on questionnaires. Birth experience must be considered of high importance when evaluating optimal treatments, and this tool provides a unique opportunity to distinguish and evaluate birth experiences.

The translation process was systematically and rigorously conducted to ensure that equivalence was established. The translation process and the cognitive interviews only resulted in minor changes in wording of the CEQ.

One concern is that item 1 of the questionnaire “The labour progress went as I had expected” does not reflect that expectations can be either negative or positive. The possible implication of the item could be that a responder with a negative labour progress replies in the same way as a responder with a positive labour progress because in both responders labour went as expected. A low score of the item may therefore not reflect a general low score of the CEQ. However we decided to retain the item since it is included in all other translated versions of the CEQ. One single item may affect the total score and the domain score (Own capacity) by 1 to 4 points prior to calculation of the mean.

Our response rate of 76.2% is comparable to the response rate in the original Swedish study (78%) and higher than the response rate in the validation studies performed in the UK (59%) and Spain (61.3%). There were very few questions not completed, suggesting that the questionnaire does not contain items that the responders find offensive or inappropriate, and therefore won’t complete.

Based on the exploratory factor analysis we found that a three-factor model was superior to the original Swedish four-factor model. The three-factor model could be viewed as a simplification of the original model, where the domains “Perceived safety” and “Own capacity” are merged into one domain except for item 18 “My impression of the medical competence made me feel secure”, which in the original Swedish model is a part of “Perceived safety” and in the Danish version is a part of “Professional support”.

A high Internal consistency was demonstrated with a Cronbach’s Alpha between 0.75 and 0.89 comparable to the Cronbach’s Alphas for all of the domains in the Swedish version of the CEQ (0.62–0.88), the UK version (0.72–0.94) and the Spanish version (0.68–0.90).

The test-retest was completed with a high attendance rate of 75%. Only the study on the UK population did also perform a test-retest, however they reported a weighted kappa and not an ICC (2,1). An ICC >0.7 for two domains and for the total score suggests that CEQ may be considered a reliable instrument when used on the two separate occasions.

Our results on known group validation concur with previous published data on other versions of the CEQ. The Swedish version found that women with long labours and operative delivery had significantly lower scores for all domains of the CEQ [9]. In the UK population, women with long labours and operative delivery reported a significantly lower score for two of the four domains of the CEQ, namely “Own capacity” and “Perceived safety” [10], corresponding to Own capacity in the three dimensional Danish version of the CEQ. The use of CEQ in parous women has also been tested in the Spanish version [11]. Our results concur with the Spanish version, where parous women reported higher CEQ score compared to nulliparous women. The Spanish population also had lower scores in women with long labours, and operative delivery [11]. We therefore conclude that the Danish version of the CEQ score is able to differentiate between women known to score differently. No other studies on the CEQ have looked at older nulliparous women. We found a tendency for such women (>35 years old) to have a lower CEQ score in all domains compared to nulliparous young women. However we did not find a significant difference in the score. This might be due to the fact that our sample of nulliparous older women was rather small (n = 48). We consider the above adequate to conclude that construct validity is acceptable.

Two domains (“Professional support” and “Participation”) showed a high ceiling effect, reducing their sensitivity to differentiate between responders. This corresponds to the finding of a large kurtosis and a skewness especially in the items of “Professional support”. This also concurs with the findings of the validation study in a Spanish population [11]. Ceiling effect were also reported for the domain “Professional support” in the Swedish version [9]. No ceiling or floor effects were reported in the UK version [10].

### Strengths

Our study sample was relatively large, which enabled us to perform additional analysis, e.g. factor analysis.

The baseline characteristics of the 128 non-responders, who did not complete the questionnaire, were similar to those of the 377 responders. We therefore assume that responders and non-responders do not differ with respect to other factors that could influence the birth experience and therefore we expect the risk of selection bias to be low.

### Limitations

The translation was performed more than one year prior to the cognitive interviews and by another group of researchers. The main author of the original Swedish CEQ was involved in the translation and all relevant documents from the process were passed on to us. However it might be considered as a limitation that all procedures were not performed or lead by the Danish research team.

Our results indicated that a three-factor model was superior to the original four-factor model. By changing the number of domains in the model it would be difficult to compare scores to other CEQ versions, which make comparisons on CEQ scores in international studies difficult.

The generalizability of our results to an unselected Danish speaking population could be affected by the fact that all responders were participants in a randomised controlled trial and received oxytocin stimulation to induce labour. About 25% of the Danish women giving birth have their labour induced[25] and only some of these are stimulated with oxytocin. One may assume that participants of this trial might have a lower score of the CEQ compared to the general population of women giving birth, due to labour induction and oxytocin stimulation.

Further, the acute caesarean section rate in our study sample was 15.3%, which is low compared to the validation studies from UK and Spain but high compared to the overall acute rate in Denmark (12.6% among nulliparous women in 2017) [26]. One might assume that the CEQ score in the general population of new mothers would be higher, since women who deliver by acute caesarean section have a lower CEQ score compared to the women who give birth vaginally, Table 6. Hence one might expect the CEQ score to be higher in the general Danish speaking population of new mothers.

## Conclusion

This study offers a transcultural adaptation of the CEQ to a Danish-speaking context. The three-dimensional Danish version of the CEQ demonstrates acceptable construct validity and reliability. We found a potential risk of interpretation difficulties in item 1, and furthermore our results revealed a significant ceiling effect, especially in the domain “Professional support”, which needs to be acknowledged when considering implementing the CEQ in trials and clinical practice.

## Supporting information

S1 Table Item scoring Danish CEQ. (PDF)

## List of abbreviations

BMI: Body Mass Index
CEQ: Childbirth Experience Questionnaire
COSMIN: COnsensus-based Standards for the selection of health Measurement Instruments
ICC: Intraclass correlation coefficient
KMO: Kaiser-Meyer-Olkin
PPH: Post Partum Haemorrhage
REDCap: Research Electronic Data Capture
UK: United Kingdom
VAS: visual analogue scale
