# Supplementary material for: The Childbirth Experience Questionnaire (CEQ)—Validation of its use in a Danish-speaking population of new mothers stimulated with oxytocin during labour
Source: PLoS One. 2020 May 14;15(5):e0233122. doi: 10.1371/journal.pone.0233122 (PMC7224492; doi:10.1371/journal.pone.0233122)
Supplement: S1 Table — (PDF) [file pone.0233122.s001.pdf]

Instructions for scoring the Childbirth Experience Questionnaire (CEQ)©

The Childbirth Experience Questionnaire (CEQ) was developed to study women's perceptions of their first labour and birth. The questionnaire comprises 22 questions and statements assessing four domains of childbirth experiences: Own capacity, Professional support, Perceived safety and Participation.

Item coding

The response format is a 4-point Likert scale ranging from Totally agree to Totally disagree. Response choices are generally coded as follows:

**Response choice**	**Coded value**
Totally agree	4
Mostly agree	3
Mostly disagree	2
Totally disagree	1

However, *ratings of negatively worded statements* (item # 3, 5, 8, 9 and 20) *are reversed* (R).

Questions about labour pain, sense of security and control (items 20-22) are assessed with visual analogue scales (VAS). The VAS-scales scores are transformed to categorical values as follows*:

**VAS score**	**Coded value**
0-40	1
41-60	2
61-80	3
81-100	4

* item 20 is reversed

Computing scale scores

Item ratings are aggregated to scale scores by summing the coded values of the items in each scale and dividing by the number of items in that scale (mean). If the respondent has answered at least half of the items in a scale then mean values of the items that have been answered should be computed. Scoring range is 1 to 4 where higher ratings reflect more positive experiences.

Reference for the CEQ: Dencker A, Taft C, Bergqvist L, Lilja H, Berg M. *Childbirth Experience Questionnaire (CEQ): development and evaluation of a multidimensional instrument.* BMC Pregnancy and Childbirth 2010, 10: 81.

For questions please contact: *anna.dencker@gu.se*

© Copyright 2011 Dencker, Taft, Bergqvist, Lilja & Berg
All rights reserved

CEQ: Domains and included items

Item number	Item	Reversed item
	Domain: Own capacity (13 items)	
1	Fødslen forløb som jeg havde forestillet mig	
2	Jeg følte mig stærk under fødslen.	
3	Jeg følte mig bange under fødslen.	R
4	Jeg følte mig i stand til at gennemføre fødslen.	
5	Jeg var træt under fødslen.	R
6	Jeg var glad under fødslen	
7	Jeg har mange positive minder fra fødslen	
8	Jeg har mange negative minder fra fødslen	R
9	En del af minderne fra fødslen kan få mig til at føle mig nedtrykt	R
19	Jeg følte, at jeg håndterede situationen godt	
20	Hvor smertefuldt oplevede du generelt fødslen?[1]	R
21	Hvor meget kontrol følte du, at du generelt havde under fødslen?[1]	
22	Når du tænker tilbage på fødslen, hvor tryg følte du dig generelt?[1]	
	Domain: Participation (3 items)	
10	Jeg følte, at jeg havde mulighed for at påvirke, om jeg skulle være oppe og røre mig eller ligge ned	
11	Jeg følte, at jeg havde mulighed for at påvirke fødselsstillingen	
12	Jeg følte, at jeg havde mulighed for at påvirke valg af smertelindring	
	Domain: Professionel support (6 items)	
13	Jordemoderen brugte tilstrækkelig tid på mig	
14	Jordemoderen brugte tilstrækkelig tid på min partner	
15	Jordemoderen informerede om, hvad der skete under fødslen	
16	Jordemoderen forstod mine behov	
17	Jeg følte, at jordemoderen behandlede mig godt	
18	Mit indtryk af sundhedspersonalets faglige kompetence gjorde mig tryg	

1. Visual analog scale
R. Item reversed in scoring
